# Colorectal cancer patients show decrease in cellular immunity and enhanced plasma level of transforming growth factor – beta and vascular endothelial growth factor (VEGF)

**DOI:** 10.1186/2051-1426-1-S1-P114

**Published:** 2013-11-07

**Authors:** Eva Zavadova, Vocka Michal, Konopasek Bohuslav, Terezie Fučíkova, Blanka Rihova, Luboš Petruzelka

**Affiliations:** 1 Department of Oncology, General Teaching Hospital and 1st Faculty of Medicine Charle’s University, Prague, Czech Republic; 2 Institute of Immunology and Microbiology, General Teaching Hospital and 1st Faculty of Medicine Charles University, Prague, Czech Republic

## Background

Colorectal cancer is the second leading cause of cancer death in the world. The cause of high mortality is that almost half the cancers are detected at an advanced stage of disease. In addition to surgical and chemotherapy treatment in recent years, applied monoclonal antibody therapy may target against growth factors, and particularly against the receptors for growth factors. In clinical practice, an antibody against vascular endotelial growth factor (VEGF) - bevacizumab-is already used. VEGF is the factor responsible for neoangiogenesis and it was considered as a possible prognostic marker of disease progression. The correlation of higher levels of VEGF with the response to applied therapy, disease progress levels and a poor prognosis in patients with solid tumors is recently discussed. Transforming growth factor - beta (Transforming growth factor - beta, TGF-beta) is also neoangiogenetic and highly immunosuppressive factor. TGF-beta suppresses natural immunity against tumors. The TGF is considered as another possible prognostic marker of disease progression.

The purpose of the study was to monitor immune responses in patients with colorectal cancer, particularly the examination of cellular as well as humoral immunity. TGF beta, and VEGF production was monitored.

## Methods

19 patients included in the research project were implemented routine cancer teratment, including bevacizumab. Basic parameters (histological type and grade, proliferative marker) were established. Patients were evaluated by a cancer clinical immunologist to exclude immune disorders, allergic or autoimmune origin. TGF-beta, VEGF were mesured by ELISA and anti-tumor cellular immunity (CD4, CD8, B cells) were measured by flow cytometry.

## Results

In patients with colorectal cancer mainly depression in cellular immunity was found. Immunglobulin plasma level was dicreased as well (mainly IgG4 subtype). Most patients have shown clinical symptoms of immunodeficiency (frequent infections of respiratory tract, herpetic infections) TGF beta as well as VEGF plasma level were increased.

## Conclusion

Correlation of these factors and the state of anticancer immunity could help in the future as a marker and contribute to the selection of targeted immune therapy in patients with colorectal cancer.

## Dedication

This project was supported by grant IGA NT 13259-3 and PRVOUK.

